# Identification of fragments targeting SMYD3 using highly sensitive kinetic and multiplexed biosensor-based screening†

**DOI:** 10.1039/d4md00093e

**Published:** 2024-03-20

**Authors:** Edward A. FitzGerald, Daniela Cederfelt, Bjarte Aarmo Lund, Nadine E. M. Myers, He Zhang, Doreen Dobritzsch, U. Helena Danielson

**Affiliations:** a Department of Chemistry – BMC, Uppsala University Uppsala Sweden helena.danielson@kemi.uu.se; b Beactica Therapeutics Virdings allé 2 Uppsala Sweden; c Science for Life Laboratory, Uppsala University Uppsala Sweden; d Department of Chemistry, UiT The Arctic University of Norway Tromsø Norway

## Abstract

A 1056-membered fragment library has been screened against SMYD3 using a novel multiplexed experimental design implemented in a grating coupled interferometry (GCI)-based biosensor. SMYD3 is a prospective target for anticancer drugs and the focus has initially been on discovery of inhibitors of its lysine methyl transferase activity. However, it has multiple protein interaction partners and several potential roles in carcinogenesis. It therefore remains unclear what mode of action ligands targeting the protein should have. Our goal was therefore to identify new ligands and discriminate hits that interact with the active site and those that interact with other sites. In addition, we were interested in selecting hits based on kinetic features rather than affinity. Screening was done in parallel against SMYD3 alone or SMYD3 with the active site blocked by a tight binding inhibitor. Hit selection was primarily based on dissociation rates. In total, 20 fragments were selected as hits, of which half apparently targeted the active site and half targeted other sites. Twelve of the hits were selected for structural analysis using X-ray crystallography in order to identify binding sites and modes of binding. Four of the hits were successfully identified in crystal structures with SMYD3; the others did not show any electron densities for ligands in the crystals. Although it might be possible to optimize the crystallography approach for a better success rate, it was clear that the sensitivity and time resolution of the biosensor assay was exceptional and enabled kinetic rate constants to be estimated for fragments. Fragments are typically considered to interact too rapidly for such quantification to be possible. This approach consequently represents a paradigm shift. In addition, the multiplexed approach allows ligands targeting different sites to be rationally selected already in the fragment library screening stage.

## Abstract 2

A 1056-membered fragment library has been screened against SMYD3 using a novel multiplexed experimental design implemented in a grating coupled interferometry (GCI)-based biosensor.

## Graphical abstract

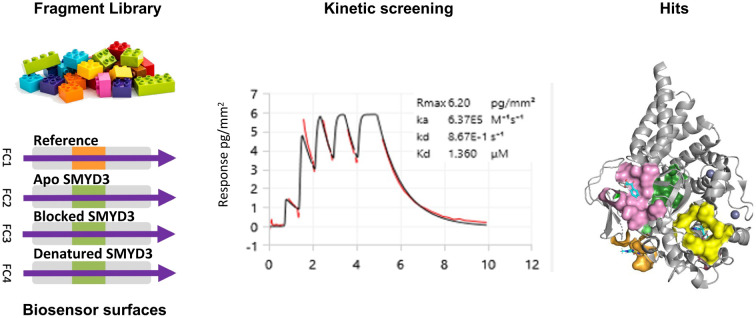

## Introduction

The drug target in this study is the epigenetic regulator SET (suppressor of variegation, enhancer of zeste, trithorax) and MYND (myeloid-Nervy-DEAF1) domain-containing protein 3 (SMYD3). This belongs to the SMYD family which consists of five proteins (SMYD1 to SMYD5) that have similar structures and domain compositions. They are lysine methyltransferases and can modify a myriad of histone and non-histone related proteins. Their major function appears to be associated with histone methylation and to a further extent chromatin remodelling and subsequent downstream gene expression.^1^ The SMYD family plays an intrinsic role in both normal and diseased states, with overexpression of SMYD2 found in cancerous tumours specifically oesophageal, bladder, and stomach whilst SMYD3 overexpression is detected in liver, colon, and breast carcinomas.^2^ However, the complete and categorical understanding of the role of SMYD3 in cancer has yet to be fully understood.^3^

We have previously established biochemical and biophysical methods for characterization of SMYD3 and its interaction with small ligands.^4^ The work led to the discovery of an allosteric site that interacts with diperodon.^5^ This subsequently led us to explore compounds selected on the basis of the diperodon structure and also perform *in silico* studies for the identification of a number of potential additional allosteric sites (FitzGerald *et al.*, manuscript). However, we were unable to probe the larger chemical space required to identify ligands interacting with the diperodon site or to the previously hypothesised sites.^5^ SMYD3 appears to be rather flexible and we found the protein to have poor stability. Consequently, for reliable experiments, conditions optimized for conformational stability, such as low temperature, are required. Here, we started anew with a novel biosensor-based approach in order to overcome the potentially insufficient sensitivity of our previously used biosensor-based assay. It allowed us to screen and identify hits in a fragment library, previously found to be useful for identifying hits to challenging targets.^6^

The use of biosensors for screening of compound libraries and the characterization of ligand–target interactions has become a routine in pharmaceutical research. The field of biosensor technology has developed since the first instruments were launched on the market. These were based on surface plasmon resonance (SPR) detection, a technology that remains very popular. However, other technologies have entered the market and are finding their niche.^7^ Generally, the new generations of instruments have higher throughput and sensitivity, as well as ease of use in drug discovery projects.

Fragment-based lead discovery (FBLD) has evolved in parallel with this technological development, and the field is currently benefiting from the highly sensitive assays that can be established for many drug targets.^8^ An advantage of using biosensors for FBLD is that state-of-the-art biosensors are useful in all stages of screening to lead optimization and can provide time-resolved data. A challenge is that fragments typically interact with low affinities and very fast kinetics. The aim is therefore typically to identify hits and rank them using equilibrium-based report points.

**Fig. 1:**
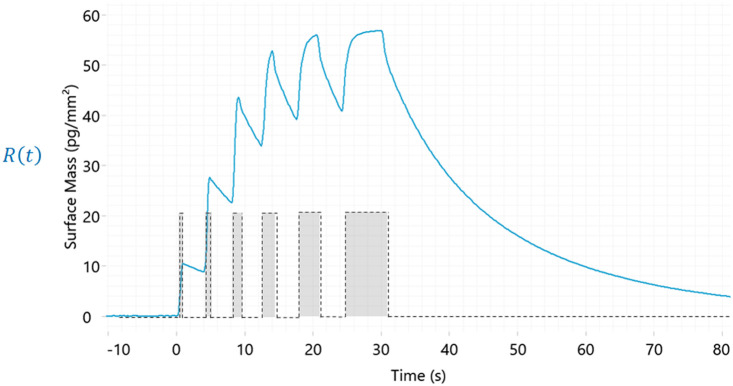
Example of data (response as a function of time) from a GCI-biosensor based kinetic experiment. The analyte is injected at a single concentration for increasing times. Complete sensorgrams encompass multiple association phases interrupted by short dissociation phases, followed by a long, final dissociation phase. Kinetic parameters are estimated from global analysis of all dissociation phases in the dataset.

Here, we explored a grating coupled interferometry (GCI)-based biosensor to overcome the challenges we have previously experienced with SMYD3. This relatively new type of biosensor technology has an integrated sensor surface and microfluidic chip that enables the rapid interactions of low affinity ligands to be resolved and hits to be identified on the basis of kinetics rather than equilibrium-based parameters. In addition, it allows an experimental design where a single concentration of analyte is injected for increasing times (Fig. 1).^9^ The same sample is used for all injections and does not require a concentration series of samples to be prepared before injection, thus reducing the time and material required for screening.

The hits identified using this novel screening approach were confirmed *via* X-ray crystallography and revealed that SMYD3 has multiple ligand binding sites, distinct from the active site that can be targeted with fragments.

## Results

### Experimental design

**Fig. 2:**
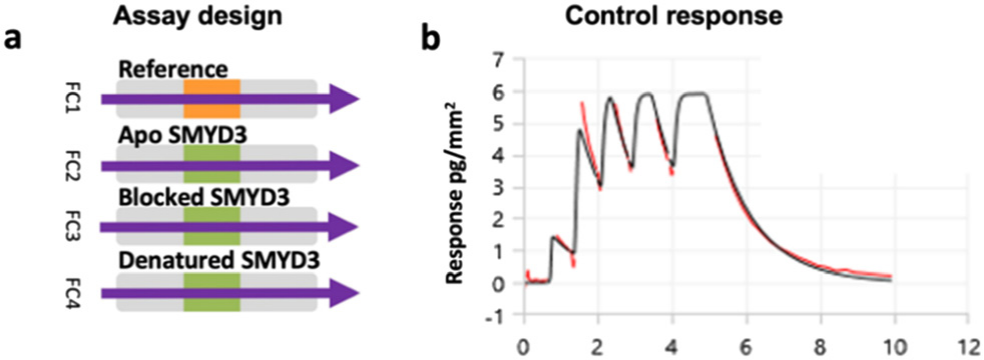
Multiplexed screening assay and sensor surface control. a) Analytes were injected into four flow channels (FC1–FC4) in parallel. b) Sensorgram for the control compound SAH (25 μM) interacting with the apo SMYD3 surface after the subtraction of the signal from the reference surface, *i.e.* FC2–FC1 signal.

A multiplexed screening assay was set up with three different SMYD3 surfaces and a blank reference surface in flow channels 1–4 (FC1–FC4) (Fig. 2a), as described previously.^5^ The apo SMYD3 surface detected fragments interacting anywhere on SMYD3, while a SMYD3 surface blocked by active site-specific tight-binding inhibitor EPZ031686 detected compounds binding elsewhere. The denatured surface served as a reference, detecting fragments binding to unfolded proteins, also potentially present to a significant degree in the other two surfaces.

After preparing the sensor surface and immobilising SMYD3, the functionality of the surface was assessed *via* analysis of interactions with the co-factor product *S*-adenosyl-homocysteine (SAH). It was used as a control in all experiments, confirming that sensor surfaces were intact. This was done using the novel multiple injection-based experimental design (Fig. 1) at 25 μM, *i.e.* in a weak binder mode (Fig. 2b).

The kinetic parameters for SAH (*k*_a_ = 3.04 × 10^5^–3.52 × 10^6^ M^−1^ s^−1^, *k*_d_ = 0.748–4.92 s^−1^) were comparable to those previously obtained using an SPR-based biosensor assay (*k*_a_ = (2.7 ± 0.1) × 10^6^ M^−1^ s^−1^, *k*_d_ = 1.6 ± 0.9 s^−1^, and the derived *K*_D_ = 611 ± 2 nM), confirming that the assay was reliable and SMYD3 was also functional in this new assay.^4^

### Kinetic screening

The library was screened using the approach described in Fig. 1, *i.e.* injection of fragments at 250 μM for increasing durations of time in a single cycle, using a flow rate of 100 μL min^−1^, data acquisition set to 40 Hz and with an association time of 5 s and dissociation of 20 s. Each fragment was addressed to all four channels and interactions with the native and blocked surfaces compared. The experimental conditions were chosen to ensure that the assay could reliably characterize the fast and transient interactions expected from typical fragments.

**Fig. 3:**
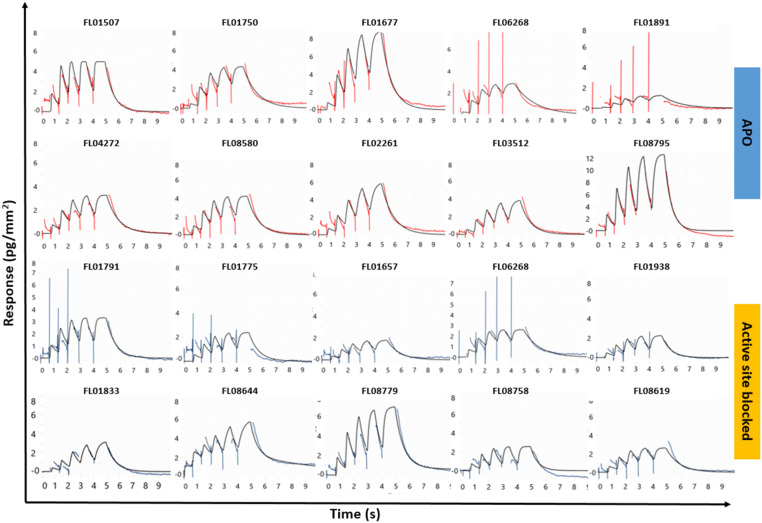
Representative sensorgrams from the kinetic screening of library FL1056 against SMYD3. The data shown are for hits selected on FC2 APO SMYD3 (top) and hits selected on active site blocked SMYD3 (bottom) and subsequently subjected to orthogonal validation *via* X-ray crystallography. A 1 : 1 interaction kinetic model was globally fitted to the sensorgrams (black traces) providing the data presented in Table 1 and plotted in the ESI,† Fig. S2.

The kinetic screening of 1056 compounds was successfully completed in 72 hours. Three primary hit calling criteria were used: 1) association and dissociation errors below 80%, 2) maximum response (*R*_max_) greater than 1.5 pg mm^−2^, and 3) hits with a *K*_D_ lower than 200 μM. Hits were identified with full kinetic information using dissociation phases. Sensorgrams for the selected hits are shown in Fig. 3 and data in Table 1.

**Table 1:** Fragment hits brought forward to orthogonal validation *via* X-ray crystallography. Apo SMYD is native SMYD3 while blocked SMYD3 is SMYD3 in complex with active site-specific tight-binding inhibitor EPZ031686. The success (+) or failure (−) of crystallization in complex with SMYD3 is specified in the XRC column. Four sites were defined (1–4, shown in Fig. 6). The interaction kinetic data (*k*_a_, *k*_d_ and *K*_D_) are from the initial screening (Fig. 3) and also plotted in the ESI† Fig. S2. The ligand efficiencies (LEs) were calculated by LE = −*RT* ln(*K*_D_)/*n*

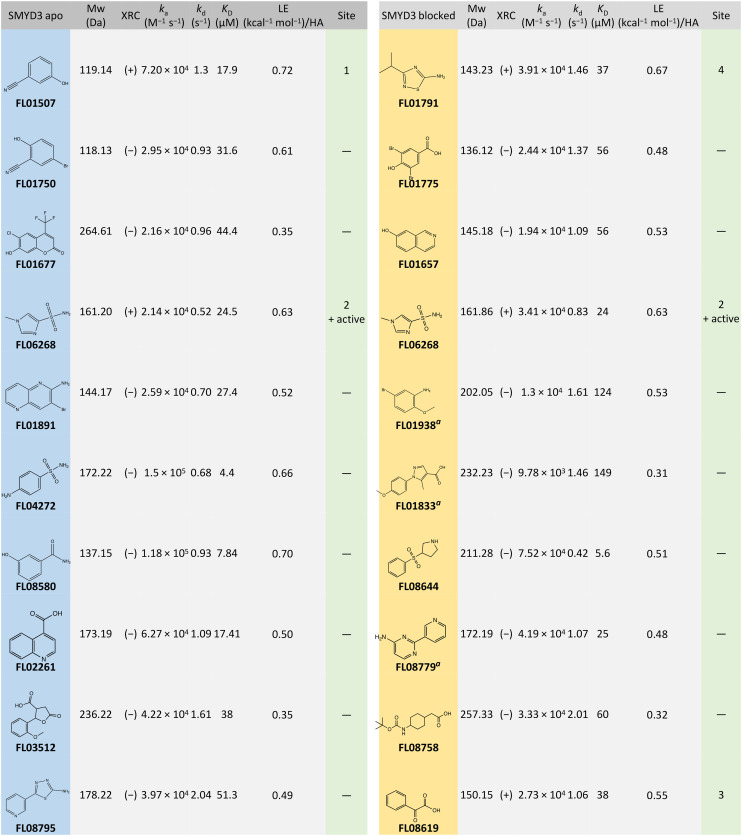

a Identified as a hit on both surfaces but did not meet the hit calling criteria on the APO surface.

After tier one hit calling, a total of 60 fragments were identified as putative hits on the surface with the apo enzyme and 75 on the surface with the blocked enzyme. The goodness-of-fit for the hits were scrutinized. When required, additional fitting and alternative kinetic models were selected. Of the 135 initial hits, 19 fragments (*i.e.* approx. 2% of the starting library) were considered for validation. Two classes of hits were selected: 1) fragments that interact preferentially with the apo enzyme (10 hits) and 2) fragments that interacted with similar binding levels to both the apo and the blocked surfaces, suggesting that they did not bind to the active site (10 hits). Interestingly, 3 fragments interacted with the blocked surface as well as the apo surface but did not pass the hit calling criteria.

**Fig. 4:**
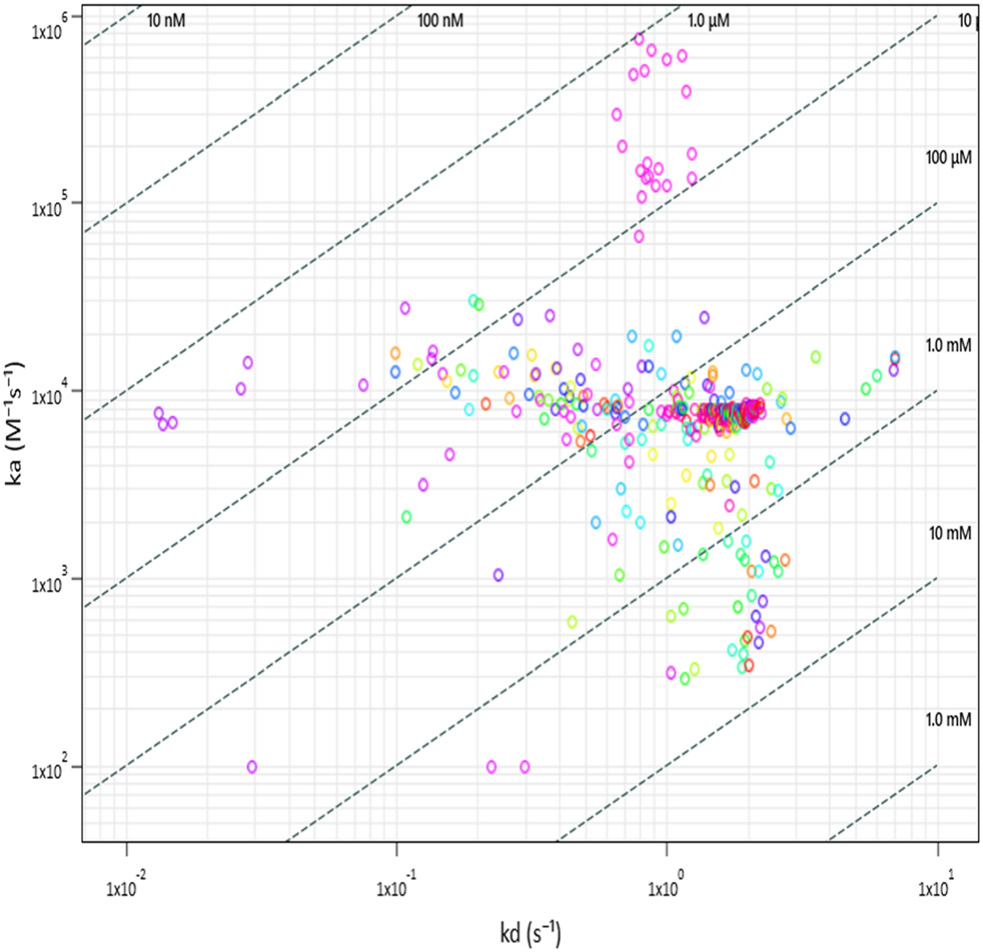
Interaction kinetic plot for 135 initial screening hits identified after applying tier 1 hit calling criteria. The data for the control compound SAH are clustered (pink).

The kinetic parameters were determined for the 135 initially selected hits and plotted in an interaction kinetic plot (Fig. 4). This illustrates the difference in kinetics for the selected fragments and shows the consistency in quantifying kinetics for the control (SAH).

### Confirmation of screening hits

Selected screening hits were also re-tested by conventional multi cycle kinetic analysis in order to confirm the interactions (some compounds were no longer available). Two variants of the GCI biosensor assay were set up, using different immobilisation levels, to allow the confirmation of hits over a broad range of affinities. The hits generally exhibited the square pulse shape typically seen for fragment interactions, but some had a strong curvature in either the association or dissociation phase, demonstrating the high time resolution of the assay (ESI,† Fig. S1a). The kinetics varied for the selected hits (ESI,† Fig. S2). All hits were not confirmed (ESI,† Fig. S1b).

### Structural analysis of screening hits

**Fig. 5:**
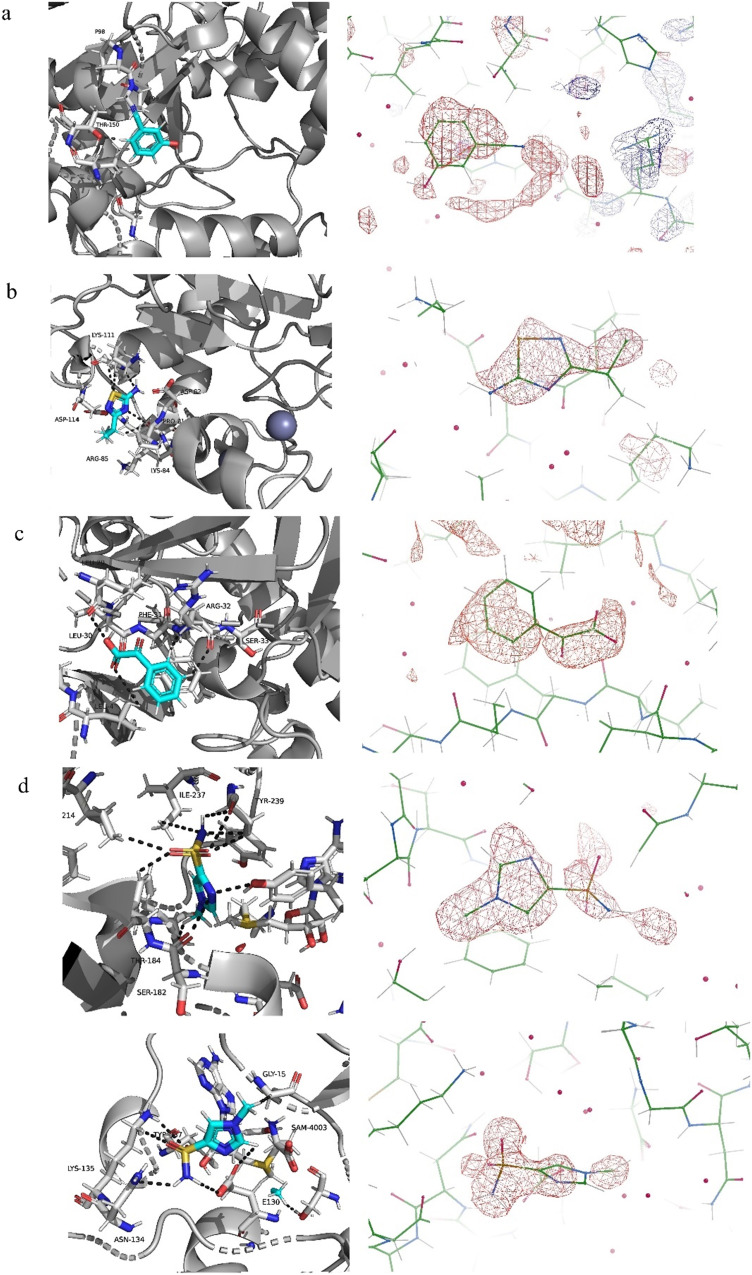
Crystal structures and (*F*_0_–*F*_c_) difference density maps (red mesh) of SMYD3 in complex with fragments (a) FL01507 (PDB-ID: 8OWO), (b) FL01791 (PDB-ID: 7QNR), (c) FL08619 (PDB-ID: 7QNU) and (d) two molecules of FL06268 (PDB-ID: 7QLB).

To identify binding sites and binding modes, the fragment hits (Table 1) were used in both co-crystallization and soaking experiments to obtain SMYD3-fragment co-crystals for X-ray crystallographic analyses. Diffraction data of 1.6–1.9 Å maximum resolution were obtained for four fragments (Fig. 5). For data collection and refinement statistics, see ESI,† Table S1.

Initially, no density features that could unambiguously be attributed to the fragments were observed in the electron density maps obtained after molecular replacement using the structure of apo-SMYD3 as a search model. Nevertheless, automated ligand fitting resulted in detection of fragments weakly bound at five different sites, of which one was the active site. Four new sites were thus identified.

Polder (*F*_0_–*F*_c_) maps (Fig. 5, right) were generated to establish whether the observed electron density features were more likely to represent background noise or to belong to bound fragments. These omit maps are generated upon exclusion of the ligand and surrounding solvent from the model, which aids in visualizing weak densities. For fragments FL01791 and FL06268, the software used for the analysis (Phenix) suggested that the omitted region was more likely a ligand than noise. For fragments FL01507, FL08580 and FL08619, a comparison of the maps obtained after refinement with a bound fragment with maps obtained upon their replacement with either glycerol or acetate, which were present in the crystallization or cryo-protectant solutions, respectively, was required. Fragments placed in electron densities fitting better to glycerol or acetate were removed from the models, and the structure obtained from SMYD3–FL08580 co-crystals was discarded since the electron density peak initially attributed to the fragment was more likely caused by glycerol.

**Fig. 6:**
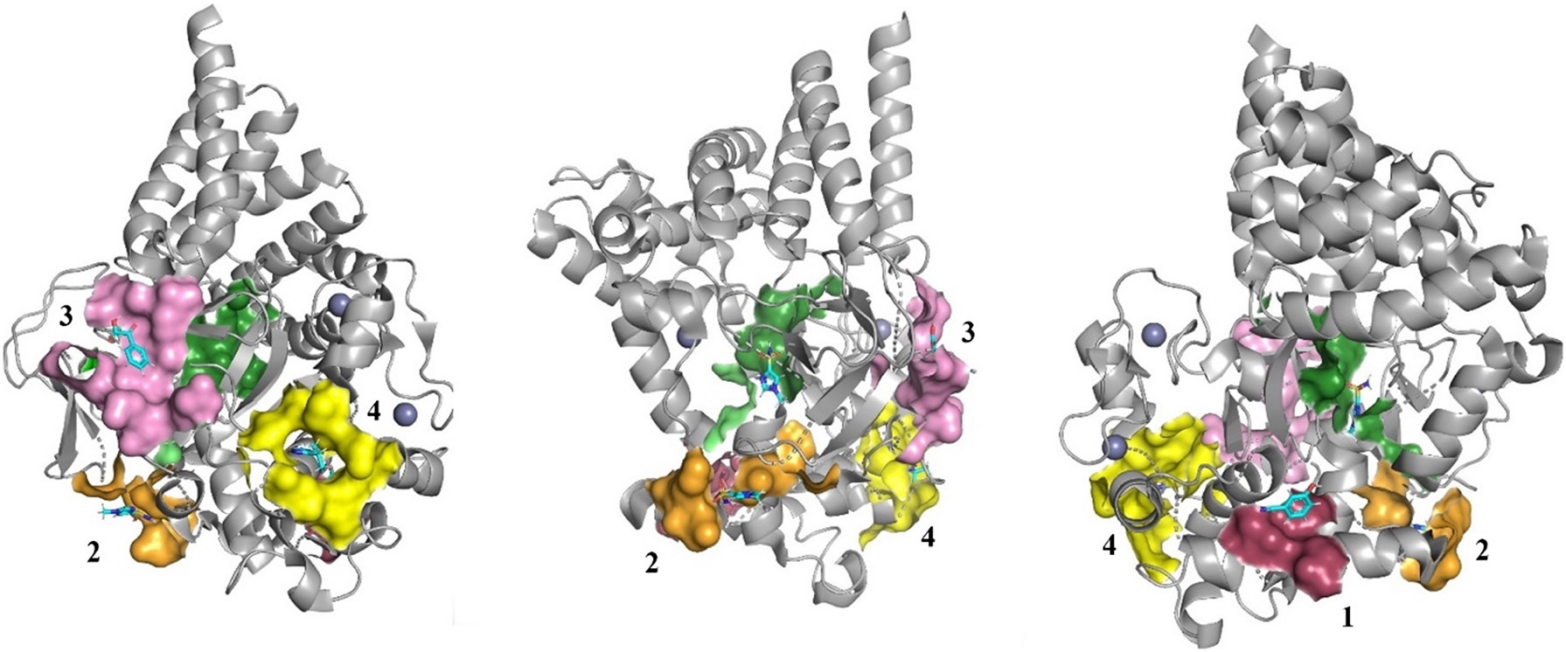
Three different views of the SMYD3 structure with newly identified ligand binding sites highlighted as surfaces and the bound fragments as stick models (coloured by atom with carbon atoms in cyan). FL01507 is bound in site 1 (red surface), FL01791 in site 4 (yellow surface), FL08619 in site 3 (pink surface), and FL06268 in both the active site (green surface) and site 2 (orange surface). The electron densities were very weak and it was difficult to establish binding modes for the fragments.

Additionally, the interactions of the fragments with residues forming the respective binding sites, illustrated in Fig. 6, were analysed using Coot. Fragments FL01791, FL08619, and FL06268 interact reasonably well with their binding sites to support our attribution of observed electron density features to them as correct. Moreover, the binding sites for FL01791 and FL08619 were previously predicted by fPocket.^5^ In contrast, only one potential hydrogen bonding interaction was identified for FL01507, and its binding site was not previously predicted, making its validity somewhat more questionable. FL06268 binds to both the active site and a site located in the SAM pocket, which was also predicted previously.^5^

## Discussion

**Fig. 7:**
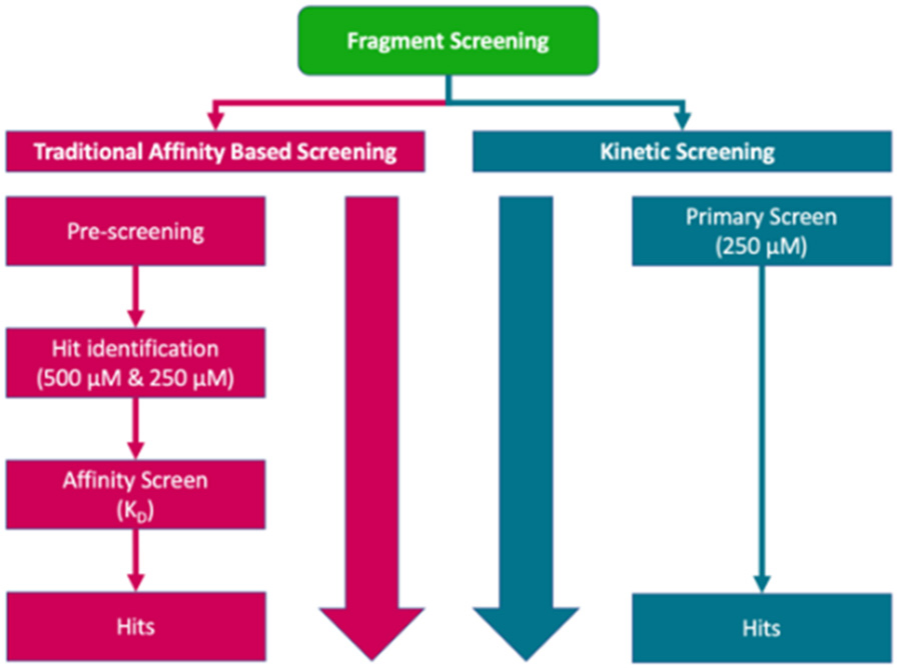
Overview of the kinetic screening workflow used and a comparison with a conventional workflow.

The transfer of the previously established SPR-biosensor assay^4^ to the GCI biosensor format was straightforward. The resulting GCI biosensor surfaces had similar properties to the SPR biosensor surfaces, as verified by similar *K*_D_ values obtained by the two methods. The multiplexing and unique experimental design available in the GCI-based biosensor was efficient for fragment library screening. The overall workflow and how it compares to conventional screening projects^6^ are illustrated in Fig. 7.

The tier 1 hit calling criteria used in the kinetic screening were selected based on statistical fitting errors *i.e.* standard deviation on the measured and fitted data for *k*_a_ and *k*_d_, followed by *R*_max_ and *K*_D_ values. An advantage of identifying hits with full kinetic information using dissociation phases is that it reduces effects of artifacts in the association phase. The hits initially identified were confirmed using conventional multi cycle analysis. Similar interaction kinetic constants and data quality were obtained in the two different experimental setups used.

Due to the low number of hits, all were taken to crystallography. If the number of compounds that can be taken to orthogonal validation needs to be reduced, additional criteria and visual inspection are recommended. Another option is to bin selected hits into different pools where each pool could be characterized by specific binding behaviours. Representative hits for each pool can then be selected for orthogonal validation.

Although the GCI biosensor analysis suggested that the hits interacted with SMYD3, the crystallography of the compounds in complex with SMYD3 was elusive. Only four fragments were successfully crystallised. There was no correlation between successful crystallisation and the interaction kinetic parameters. Moreover, the electron densities of the crystallisable hits were weak.

The possibility to multiplex the screen and identify fragments interacting with different sites is an important feature of the current screening approach. Fragments were thus identified for 4 previously predicted binding pockets.^5^ Moreover, the hit selection criteria were useful but included compounds that were not easy to progress *via* structural data. The results suggest that the new strategy for fragment library screening is powerful and the possibility to focus on the interaction kinetic characteristics of fragments for selection of compounds is likely to be suited for structure-based evolution. Seeing that the screening can be done within a few days makes it very attractive as an approach for identifying hits.

FL01791 and FL08619 are considered to be the most reliable starting points for generation of ligands with improved interaction properties. They bind in previously predicted sites, distinct from the SAM-binding pocket and with a clear electron density. However, the druggability scores for these predicted binding sites were low. Fragment FL06268 had a reliable electron density but was found binding in the SAM pocket. Fragment FL01507 is considered the least reliable starting point for generation of new ligands, since the associated electron density was not as clear and the binding site had not been predicted.

These fragments represent novel starting points for evolution of tool compounds that can interfere with interactions between different sites of SMYD3 and other proteins. However, further work is clearly required to evolve the fragments into ligands amenable to a structure-based approach and to explore their interactions with the newly identified binding sites.

## Experimental

### Chemicals

*S*-Adenosyl-methionine (SAM), *S*-adenosyl-homocysteine (SAH), EPZ031686 (6-chloro-2-oxo-*N*-((1*R*,3*r*,5*S*)-8-(((1-(4,4,4-trifluorobutyl)piperidin-4-yl)methyl)sulfonyl)-8-azabicyclo[3.2.1]octan-3-yl)indoline-5-carboxamide), 2-amino-2-(hydroxymethyl)-1,3-propanediol (Tris), 2,2-bis(hydroxymethyl)-2,2′,2′′-nitrilotriethanol (Bis-Tris), sodium chloride (NaCl), Tween 20, 4-(2-hydroxyethyl)piperazine-1-ethanesulfonic acid (HEPES), dimethyl sulfoxide (DMSO), dl-dithiothreitol (DTT), magnesium chloride (MgCl_2_), and polyethylene glycol 3350 (PEG3350) were from Sigma-Aldrich.

### Fragment library

The fragment library was comprised of 1056 fragments collated from compound collections at SciLifeLab^10^ and FRAGNET (https://fragnet.eu/, a Marie Skłodowska-Curie Action Innovative Training Network (ITN) 2016–2020).^6^ Fragments were selected on the basis of key physicochemical properties, including heavy atom count (HAC), molecular weight (MW) and calculated lipophilicity (cLog *P*). The selection criteria essentially matched the guidelines put forward by Astex for typical fragments (MW < 300 Da, cLog *P* < 3, hydrogen bond acceptors (HBA) ≤ 3, hydrogen bond donors (HBD) ≤ 3).^11,12^ The FragNet collection includes 3D fragments, *i.e.* compounds that are not flat, but with a more complex structure.^13^

### SMYD3 production and purification

Full length recombinant SMYD3 was produced as previously reported.^4^ Briefly, the protein was overexpressed in *E. coli* BL21(DE3), in some cases the Rosetta 2 strain. The protein was purified by immobilized metal affinity chromatography (IMAC), followed by tag cleavage with thrombin, reverse IMAC, and anion exchange chromatography. Fractions containing pure protein were concentrated to 8 mg mL^−1^ in 50 mM Tris-HCl (pH 8.0), 150 mM NaCl, and 2 mM DTT buffer. The homogeneity of the isolated protein was estimated from SDS-PAGE to approx. 95%, with an average yield of 5 mg of the pure protein from 1 L of culture.

### Biosensor assay

All interaction kinetic experiments were conducted with a GCI–flow-based biosensor (WAVEdelta, Creoptix AG/Malvern Panalytical) and PCH WAVEchip (Creoptix AG/Malvern Panalytical) sensor chips. The sensor chips were conditioned using injections of borate buffer (10 mM sodium tetraborate pH 8.5, 1 M NaCl). The running buffer composition, if not otherwise stated, was TBS buffer (50 mM Tris, 150 mM NaCl, 0.5 mM TCEP, 0.05% Tween 20, 1% DMSO, pH 8). SMYD3 was diluted to the desired concentration in 10 mM bis-Tris (pH 7.0) and amine coupled to the sensor surface of the PCH sensor chip. The sensor chip was functionalized at 25 °C by immobilising SMYD3 with EDC and NHS (Xantec) using an injection time of 420 s and a flow rate of 10 μL min^−1^. The immobilisation levels ranged from 5000 pg mm^−2^ to 18 500 pg mm^−2^ surface mass to enable analysis of fragments spanning a broad range of affinities. After immobilization, the surface was deactivated with a running buffer containing 50 mM Tris for 420 s. The GCI data referencing and analysis were performed using WAVEcontrol V4.5 (Creoptix AG).

Kinetic measurements with controls and fragments were performed at 15 °C. Analysis of controls and follow up of hits was done using the multi cycle kinetic (MCK) injection of a concentration series for the same time, with a two-fold serial dilution starting at 250 μM for each compound. Solvent correction was performed ranging from 0.5–1.8% DMSO. Blank samples of the running buffer, 1× TBS (50 mM Tris, 150 mM NaCl, 0.5 mM TCEP, 0.05% Tween 20, 1% DMSO, pH 8), were injected during the measurements every fifth cycle. The experimental design illustrated in Fig. 1 was used for the screening against all four surfaces. The sensorgrams were adjusted to account for solvent correction and blank subtraction. Kinetic fitting was performed with the Direct Kinetics engine of WAVEcontrol software V4.5 (Creoptix AG) with a suitable fitting model.

### Crystallization of SMYD3–fragment complexes

8 mg mL^−1^ of SMYD3 in storage buffer (TBS buffer containing 2 mM DTT) was pre-incubated for 4–8 hours with 5 mM of a respective fragment hit compound and 10% (v/v) DMSO. Subsequently, 1 μL of this solution was mixed with an equal volume of reservoir solution (100 mM Tris, 100 mM magnesium acetate, 15–17% PEG3350, pH 8.25–8.75) to create the drops for co-crystallization by hanging drop vapour diffusion performed at 20 °C. Needle-like co-crystals nucleated within 12 h of equilibration against the reservoir. In addition, crystals of SMYD3 obtained in ligand-free crystallization setups were soaked with fragment solution prior to freezing. All crystals were cryo-protected *via* brief immersion in reservoir solution supplemented with 10% (v/v) glycerol.

Diffraction data were collected at beamline ID23-1 of the European Synchrotron Radiation Facility (ESRF, Grenoble, France). Data were indexed, auto-processed, scaled and merged on-site using the implemented data processing routines and software. Fragment-associated electron density features were identified upon data analysis with the Pipedream system (version 1.4.0, Global Phasing Ltd, Cambridge, United Kingdom), which includes data processing with autoPROC,^14^ molecular replacement with Phaser,^15^ structure refinement with BUSTER version 2.10.4 (Global Phasing Ltd, Cambridge, United Kingdom), automated ligand fitting with Rhofit (Global Phasing Ltd, Cambridge, United Kingdom) and BUSTER post-refinement.^16^ Refinement was done with Phenix,^17^ and model building with Coot.^18^ (*F*_0_–*F*_c_) difference density maps for models, from which ligand atoms and surrounding water molecules were removed, were generated using Phenix.

## Conclusions

The GCI biosensor with technical features and the new assay design used here are faster and have a higher kinetic resolution than conventional assays. It was confirmed that fragments indeed interact with different association and dissociation rates and that relevant kinetic parameters can be quantified at the stage of screening.

X-ray crystallography played a pivotal role in confirming some of these hits as *bona fide* hits and validates that the chosen approach represents a good proof of principle. However, the extremely fast fragments may fall outside the sensitivity of XRC, which emphasizes the advantage of highly sensitive time resolved biosensor-based assays.

## Abbreviations

GCI: Grating coupled interferometry
SAH: *S*-Adenosyl-homocysteine
FBLD: Fragment-based lead discovery
FC#: Flow channel number
*R*_max_
: Maximum response
SPR: Surface plasmon resonance

## PDB ID codes

Authors will release the atomic coordinates and experimental data upon article publication. 8OWO, 7QLB, 7QNR and 7QNU.

## Author contributions

Edward A. FitzGerald: GCI biosensor experiments and study design. Daniela Cederfelt: production of SMYD3 and X-ray crystallography. Bjarte Aarmo Lund: X-ray crystallography. Nadine E. M. Myers: production of SMYD3 and GCI biosensor experiments. He Zhang: production of SMYD3 and GCI biosensor experiments. Doreen Dobritzsch: supervision of X-ray crystallography. U. Helena Danielson: project leader, supervision and overall interpretation of results.

## Conflicts of interest

There are no conflicts to declare.

## Supplementary Material

MD-015-D4MD00093E-s001
