# Supplementary material for: Identification of fragments targeting SMYD3 using highly sensitive kinetic and multiplexed biosensor-based screening
Source: RSC Med Chem. 2024 Mar 20;15(6):1982–90. doi: 10.1039/d4md00093e (PMC11187542; doi:10.1039/d4md00093e)
Supplement: MD-015-D4MD00093E-s001 [file MD-015-D4MD00093E-s001.pdf]

Electronic Supplementary Material (ESI) for RSC Medicinal Chemistry.
This journal is © The Royal Society of Chemistry 2024

Supplementary data

Identification of fragments targeting SMYD3 using highly sensitive kinetic and multiplexed biosensor-based screening

Edward A. FitzGerald,[a,b] Daniela Cederfelt,[a] Bjarte Aarmo Lund,[a,d] Nadine E. M. Myers[a,b] He Zhang,[a] Doreen Dobritzsch[a] and U. Helena Danielson[a,c] *

[a] *Department of Chemistry - BMC, Uppsala University, Uppsala, Sweden.*
[b] *Beactica Therapeutics, Virdings allé 2, Uppsala, Sweden.*
[c] *Science for Life Laboratory, Uppsala University, Uppsala, Sweden.*
[d] *Department of Chemistry, UiT The Arctic University of Norway, Tromsø, Norway.*
* Corresponding author: helena.danielson@kemi.uu.se

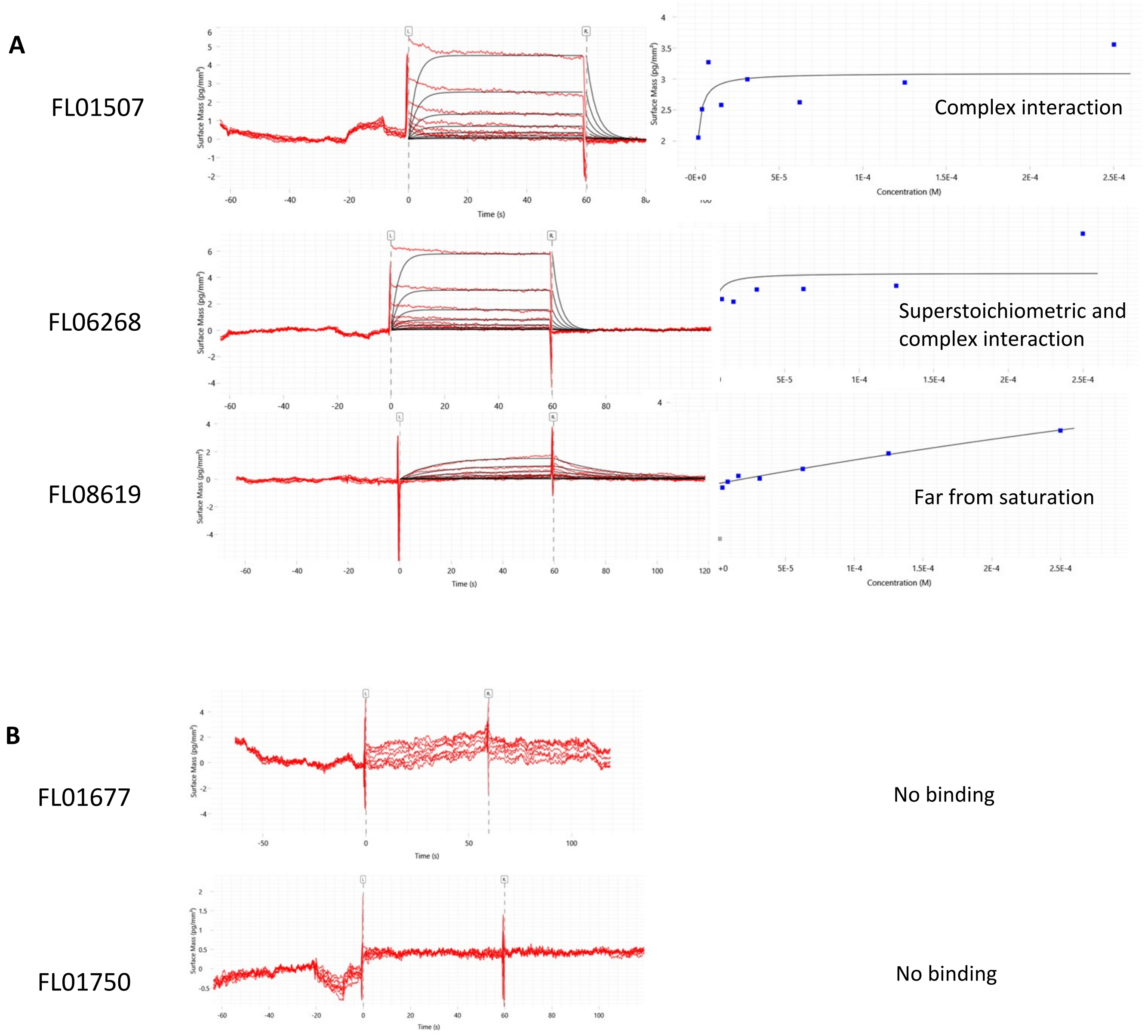

Figure 1. Examples of sensorgrams from multicycle kinetic experiments with initial fragment hits and SMYD3. A) Fragments for which crystal structures of the complex with SMYD3 were obtained. A global fit of the sensorgrams using a 1:1interaction model shows that the interactions have low affinity and are complex. B) Fragments for which crystal structures were not obtained.

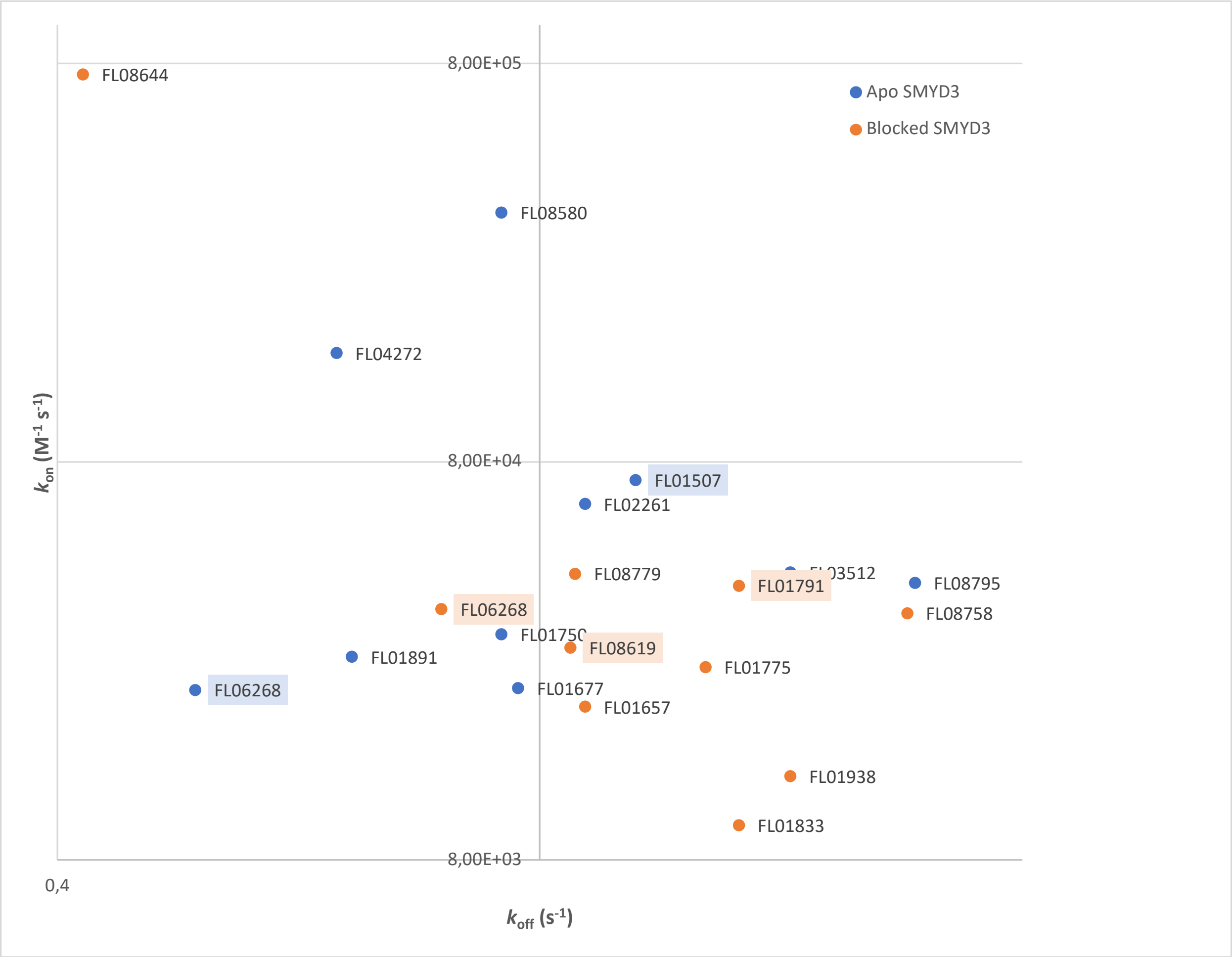

Figure 2. Interaction kinetic plot for initially selected hits, based on data from kinetic screen (Table 1). Fragments that were successfully crystallised have shaded labels.

Table 1: Data collection and refinement statistics for crystal structures of SMYD3 with fragment hits. Values in brackets are for the outer resolution shell.

**Fragment**	**FL01507**	**FL01791**	**FL08619**	**FL06268**
PDB accession code	8OWO	7QNR	7QNU	7QLB
**Data collection**				
X-ray source	ESRF ID23-1	ESRF ID23-1	ESRF ID23-1	ESRF ID23-1
Space group	$P\ 2_1\ 2_1\ 2_1$	$P\ 2_1\ 2_1\ 2_1$	$P\ 2_1\ 2_1\ 2_1$	$P\ 2_1\ 2_1\ 2_1$
Wavelength (Å)	0.9763	0.9655	0.9763	0.9763
Unit cell (Å°)	61.1 66.1 107.3 90 90 90	60.2 66.2 105.3 90 90 90	59.9 65.9 105.1 90 90 90	60.9 66.3 108.2 90 90 90
Resolution range (Å)	53.08-1.80 (1.86-1.80)	44.56-1.57 (1.63-1.57)	41.08-1.64 (1.70-1.64)	29.33 - 1.80 (1.86 - 1.80)
Unique reflections	40369 (3515)	58248 (5660)	48981 (149)	41050 (3767)
Multiplicity	12.8 (8.9)	4.0 (4.0)	6.2 (4.6)	2.0 (2.0)
Completeness (%)	98.4 (87.5)	97.8 (96.3)	75.0 (2.9)	99.2 (93.2)
R_{meas}	0.0524 (0.2858)	0.1531 (2.967)	0.2339 (3.425)	0.0270 (0.2042)
$I/\sigma(I)$	31.18 (6.47)	7.03 (0.50)	6.45 (0.49)	24.10 (4.87)
CC(1/2)	1 (0.976)	0.996 (0.171)	0.991 (0.134)	0.999 (0.95)
**Refinement**				
R_{work}	0.1555 (0.1668)	0.2036 (0.3746)	0.1855 (0.3124)	0.1506 (0.1913)
R_{free}	0.1810 (0.1972)	0.2339 (0.3429)	0.2296 (0.2889)	0.1834 (0.2308)
Average B-factor ($Å^2$)	29.02	29.62	23.61	27.51
Protein	28.16	29.12	22.60	26.43
Ligands	45.54	27.46	22.50	35.18
Solvent	34.64	34.94	31.50	34.06
No. non-hydrogen atoms	3973	3822	3925	4003
Protein	3470	3443	3439	3443
Ligands	18	67	66	86
Solvent	482	340	445	510
r.m.s.d.				
bond lengths (Å)	0.012	0.006	0.009	0.011
bond angles (°°)	1.35	0.84	0.88	1.05
Ramachandran plot, residues in				
favored region (%)	98.11	97.64	98.35	98.11
allowed region (%)	1.89	2.36	1.65	1.89
outlier region	0.00	0.00	0.00	0.00
